# A Monument to the Memory of the Late Dr. Willis F. Westmoreland, Sr.

**Published:** 1893-09

**Authors:** B. W. Allen

**Affiliations:** Hurtsboro, Ala.


### *CORRESPONDENCE.*

### A MONUMENT TO THE MEMORY OF THE LATE DR. WILLIAM F. WESTMORELAND, SR.

We print below a letter from Dr. B. W. Allen, upon this subject. It is a matter for consideration by the alumni of the Atlanta Medical College, who listened to the teachings of this famous and beloved professor. THE JOURNAL will be glad to print any communications upon the subject of a monument to be erected by contributions from the alumni.

HURTSBORO, ALA., August 17, 1893.

*Editor Atlanta Medical and Surgical Journal:*

SIR—In conversation to-day with Drs. W. B. Prather and R. N. Pitts, the suggestion was incidentally made that the old students of the Atlanta Medical College who graduated under the teachings of Dr. Willis Westmoreland ought to erect a monument to his memory. After thinking the matter over it occurred to me to write your journal with the request that you publish this and see what the old students think of the advisability of the undertaking. Hill, Stephens and Grady have monuments erected to their memory on account of their statesmanship. Now, why not erect one to the memory of Georgia's greatest surgeon, not only the greatest, but one who untiringly strove to teach his students correct principles of surgery? Now, what say the alumni of the Atlanta Medical College? I would be glad to hear from any who may chance to see this with regard to the undertaking. Hoping that you will give this space in your journal, I beg to remain

Yours truly,

B. W. ALLEN.

---

IT is a common idea among the laity that a persistent pain in the small of the back means kidney trouble. As a matter of fact, acute nephritis from cold is the only kidney disease that announces itself to the patient by pain.—*Baumgarten.*

